# Clinical Features and Ultrasonographic Manifestations of Retroperitoneal Nerve Sheath Tumors

**DOI:** 10.2174/0115734056348636241213120140

**Published:** 2025-01-02

**Authors:** Xiaoqing Wang, Xiaoying Zhang, Rui Zhao, Yan Liu, Chaoyang Wen, Haining Zheng

**Affiliations:** 1 Department of Ultrasound, Peking University International Hospital, Beijing, China; 2 Department of Pathology, Peking University International Hospital, Beijing, China

**Keywords:** Retroperitoneal nerve sheath tumors, Ultrasonography, Diagnosis, Tumor characterization, Malignant potential, Patient outcomes

## Abstract

**Objectives:**

Retroperitoneal nerve sheath tumors are uncommon, representing a small fraction of all primary retroperitoneal neoplasms. Accurate differentiation between benign and malignant forms is essential for optimal clinical management. This study assessed the clinical profiles and sonographic traits of retroperitoneal nerve sheath tumors with the goal of enhancing diagnostic precision and developing therapeutic strategies.

**Methods:**

A retrospective analysis of patients diagnosed with retroperitoneal nerve sheath tumors who completed surgical treatment and underwent ultrasound imaging was carried out. Tumors were classified based on sonographic features and blood flow characteristics as per Adler's grading system. Statistical analysis was performed using SPSS 25.0. ROC curve analysis was carried out to determine the optimal diagnostic cutoff values.

**Results:**

A total of 57 patients were included in the study. There were no significant variances in age, gender, or tumor localization among the groups. However, pronounced disparities were observed in tumor number, size, shape, definition of borders, internal echo pattern, structural composition, presence of calcification, and blood flow signals between the classic and malignant groups. Notably, malignant tumors tended to manifest as larger masses with indistinct margins and irregular shapes. The maximum tumor diameter emerged as a discriminating factor for malignancy, with a diagnostic cutoff of 9.9 cm, yielding an AUC of 0.754 from the ROC curve analysis.

**Conclusion:**

This study outlines the distinctive clinical and sonographic features of retroperitoneal nerve sheath tumors, with a particular focus on malignant subtypes. Ultrasonography emerges as a valuable diagnostic tool, contributing to the differentiation of tumor categories and potentially to the development of targeted treatment strategies. The identification of specific sonographic markers may facilitate the early detection and detailed characterization of these tumors, which could contribute to improved patient outcomes.

## 1 INTRODUCTION

Retroperitoneal nerve sheath tumors are a relatively uncommon group of malignancies within the retroperitoneal space, with an estimated incidence of 0.7% to 2.7% among all primary retroperitoneal neoplasms [1-3]. The majority of these tumors are benign; however, malignant peripheral nerve sheath tumors (MPNSTs) are exceedingly rare, with a reported incidence of 0.001% [4-6]. MPNSTs include malignant schwannomas, neurogenic sarcomas, malignant neuri- lemmomas, and neurofibrosarcomas. Nerve sheath tumors can be classified into classic and cell-rich subtypes, and discerning their clinical and radiographic features is essential for precise diagnosis and treatment. The current clinical research on retroperitoneal nerve sheath tumors is limited, especially in distinguishing between classic and cell-rich subtypes. Therefore, there is a lack of literature on malignant variants.

On radiology, nerve sheath tumors display the relationship to the nerve, fusiform shape, “split-fat” sign, and associated muscle atrophy. MPNSTs involve the major nerve trunks and tend to be greater than 5cm. They may have ill-defined margins, suggesting infiltration of adjacent tissues and edema [7]. Ultrasonography is a cost-effective and reliable tool for assessing peripheral nerve sheath tumors [8].

Our research aims to address this knowledge gap by providing an in-depth analysis of the epidemiology, clinical features, and sonographic profiles of retroperitoneal nerve sheath tumors. We also aim to establish a diagnostic framework that effectively distinguishes among the classic, cell-rich, and malignant subtypes. This may aid in accurate classification and optimal therapeutic planning for these rare tumors.

## 2 MATERIALS AND METHODS

### 2.1 General Information

This study involved a comprehensive retrospective analysis on 57 individuals diagnosed with retroperitoneal nerve sheath tumors between April, 2015 and July, 2023. Inclusion criteria were based on the completion of surgical treatment, availability of comprehensive postoperative pathology reports, and access to complete ultrasound imaging data.

### 2.2 Instruments and Methods

For the ultrasonographic evaluation, we employed a state-of-the-art Philips IU22 and a MINDRAY Resona 7S color Doppler ultrasound systems, integrating a standard C5-1 abdominal convex array probe for general imaging and an L12-5 linear array probe for detailed assessments when necessary.

The tumors were thoroughly examined and classified according to a range of criteria: their anatomical location (abdominal, pelvic, or a combination of both), number (single or multiple), size, shape (regular or irregular), border definition, internal echogenicity patterns (hypoechoic, isoechoic, or mixed), calcification presence, tissue demarcation, and blood flow characteristics as per Adler's grading system.

Conducted as a retrospective study, our research was approved by the Ethics Committee of Peking University International Hospital, which granted a waiver for the requirement of informed consent. All identifiable information was removed or anonymized to ensure individual patients could not be identified. The data was stored securely in password-protected and encrypted databases, with access limited to authorized research personnel only. The study was conducted in strict accordance with the ethical standards as outlined in the Declaration of Helsinki by the World Medical Association.

### 2.3 Statistical Analysis

Data analysis was performed utilizing SPSS 25.0 software. The normality of the data was assessed using the Shapiro–Wilk test. According to the Shapiro-Wilk test (SW test), the age of the highly cellular type and the maximum diameter of the classic type both deviated from a normal distribution. Therefore, non-parametric tests were used in the study. Continuous variables were analyzed through the Kruskal-Wallis test. Categorical variables, presented in percentages, were evaluated using chi-square tests or Fisher's exact tests, depending on the context. Additionally, the ROC curve was delineated to ascertain the area under the curve (AUC) and determine the optimal cutoff value for diagnostic accuracy. Furthermore, multiple regression analysis was carried out to account for potential confounding factors. Statistical significance was defined by a *p*-value of less than 0.05.

## 3 RESULTS

The study population was categorized into two distinct groups: the first group consisted of 36 classic-type cases with a gender ratio of 12 males to 24 females, encompassing a broad age spectrum from 15 to 74 years; the second group included 13 cell-rich type cases with a gender distribution of 4 males to 9 females, with ages spanning from 32 to 65 years. Furthermore, our analysis identified a subset of 8 patients with malignant retroperitoneal nerve sheath tumors, comprising 5 males and 3 females, with ages ranging from 21 to 49 years.

### 3.1 Analysis of Clinical Data

In our retrospective investigation, we dissected the clinical and oncological profiles of individuals with classic, cell-rich, and malignant peripheral nerve sheath tumors, as detailed in Table **1**. Of particular interest was the mean age among patients with malignant variants, which trended lower; however, this age variance did not reach statistical significance across the groups (*p*=0.172). Paralleling this, no discernible sex distribution disparity was identified between the groups (*p*=0.273).

A poignant observation was the predilection for solitary tumors in the classic and cell-rich categories, in contrast to the malignant category, which exhibited a pronounced prevalence of multiplicity, indicating a statistically significant variance (*p*=0.004). The distribution of tumor locales, abdominal, pelvic, or abdominopelvic, demonstrated equitable dispersion among the three groups, with no statistically significant differences (*p*=0.113).

The results of multiple regression analysis are given in Table **3**. The multiple regression analysis for the highly cellular group did not yield statistically significant predictors for tumor characterization. Gender showed a non-significant association with tumor type (OR: 2.250, 95% CI: 0.442–11.446; *p*=0.329). Similarly, age had a weak association with the outcome (OR:1.044, 95% CI: 0.990–1.100; *p*=0.113). Maximum diameter also showed no significant association (OR:1.006, 95% CI: 0.823–1.229; *p*=0.954). Morphology showed a potential trend (OR: 13.328, 95% CI: 0.618–287.465; *p*=0.098) but did not reach statistical significance.

### 3.2 Ultrasonic Characterization

**Fig. (1A-C):**
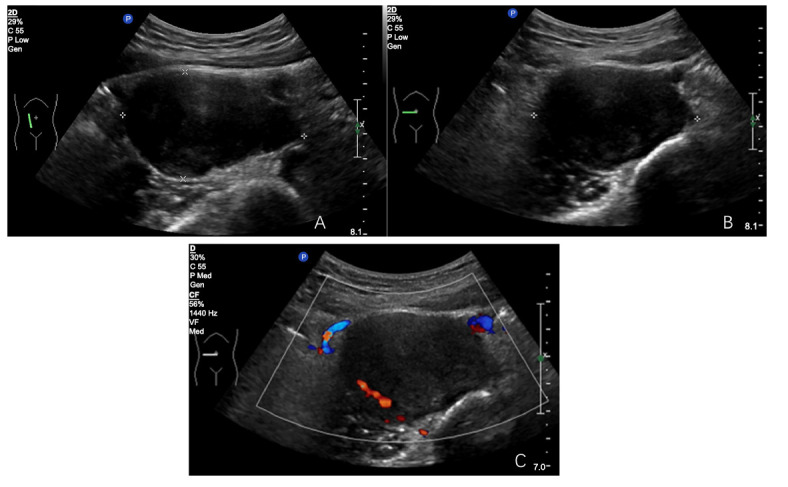
Classic retroperitoneal nerve sheath tumor located in the right anterior aspect of the right abdominal spine, with well-defined borders, regular shape, and grade 2 blood flow signals seen within.

**Fig. (2A-C):**
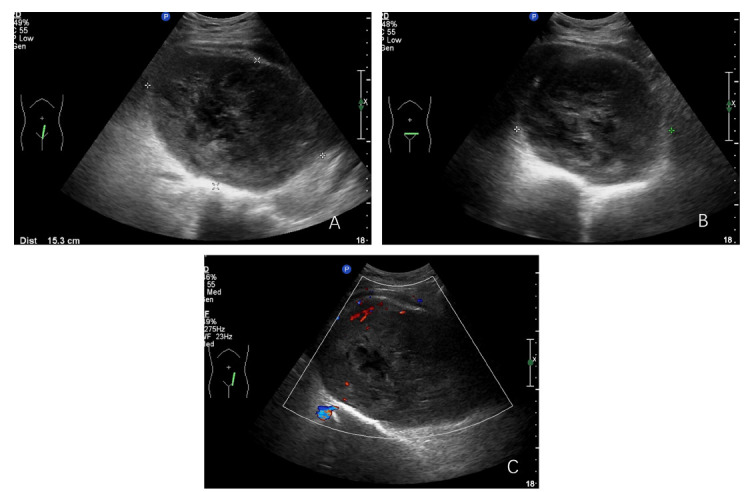
Malignant retroperitoneal nerve sheath tumor, located anterior to the pelvic spine, with poorly defined borders, irregular morphology, heterogeneous internal echogenicity, and grade 2 blood flow signal.

**Fig. (3A-C):**
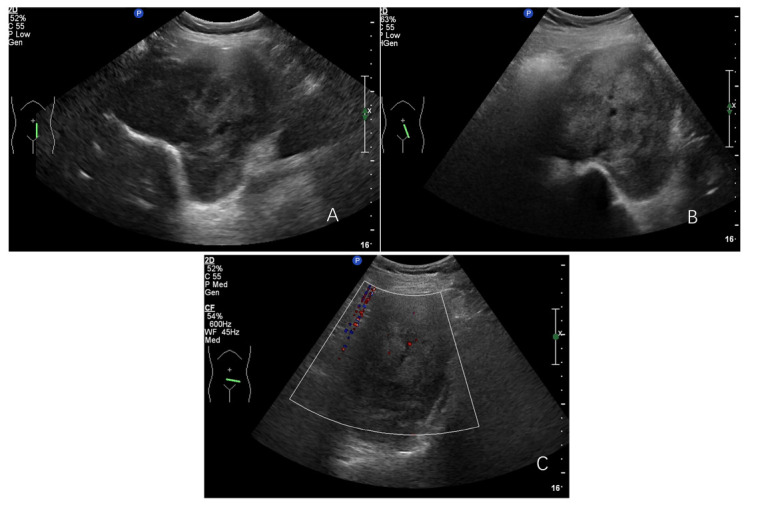
Cell-rich retroperitoneal nerve sheath tumor, located anterior to the left lower abdominal spine, with well-defined, lobulated borders and grade 1 blood flow signals seen within.

In our retrospective assessment of tumor ultrasound characteristics, we identified pronounced disparities in tumor dimensions, with the malignant cohort presenting a notably larger mean maximum diameter when juxtaposed with the classic and cell-rich groups (*p*=0.054), as mentioned in Table **2**. The contours and form of malignant nerve sheath tumors were distinctly atypical, characterized by indistinct borders (*p*=0.000) and a predominance of irregular shapes (*p*=0.001), which were in stark contrast to the other tumor types (Figs. **1**-**3**).

While there were observable variations in internal echogenicity, composition, calcification, and blood flow grading among the groups, these did not attain statistical significance (*p*=0.115). Nonetheless, the delineation of malignant nerve sheath tumors from adjacent tissues was significantly less distinct compared to the other groups (*p*=0.000).

The ultrasound visualizations of malignant nerve sheath tumors were typified by indistinct margins and irregular forms, reflective of their invasive properties. The heterogeneity in internal echoes and irregularity in the margins are further indicators of their malignancy.

### 3.3 ROC Curve Analysis

Fig. (**4**) illustrates the ROC curve analysis, which discerns a notable distinction in the maximum tumor diameter across the three groups. The AUC of the ROC curve was calculated to be 0.754 (95% CI: 0.573–0.934), demarcating a diagnostic threshold for maximum tumor diameter at 9.9 cm. This threshold indicates that tumors exceeding this measurement may warrant further consideration for malignancy.

## 4 DISCUSSION

We examined the clinical and oncological profiles of patients with classic, cell-rich, and malignant peripheral nerve sheath tumors. Classic and cell-rich tumors were often found to be solitary, while malignant tumors showed a higher prevalence of multiplicity (*p*=0.004). ROC curve analysis indicated a significant difference in maximum tumor diameter among the groups, with an AUC of 0.754 (95% CI: 0.573–0.934), establishing a diagnostic threshold of 9.9 cm for potential malignancy. Tumors exceeding this size should prompt further evaluation for malignancy.

Peripheral nerve sheath tumors are characterized by neoplastic proliferations with Schwann cell differentiation. Unlike classical schwannomas, retroperitoneal cellular schwannomas commonly express GFAP and CK, suggesting that they may originate from unmyelinated Schwann cells, unlike classical schwannoma, which may originate from myelinated Schwann cells [9]. Surgical resection is the mainstay treatment strategy to increase survival in localized MPNSTs. Radiotherapy is commonly administered to improve local control, and neoadjuvant administration of radiotherapy is increasing in popularity as it decreases radiation dosage [10]. Prompt diagnosis is necessary for earlier intervention that improves diagnosis. Nerve sheath tumors are predominantly encountered in the head, neck, and extremities, with occurrences in the retroperitoneal region being relatively rare [11, 12]. The distribution of tumor locations across abdominal, pelvic, and abdominopelvic regions was similar among the groups in our study. These retroperitoneal neoplasms, although infrequent, exhibit a close affinity to the nerves of the retroperitoneal space, often manifesting in areas, such as the paraspinal region, renal hilum, and presacral pelvic regions, where nerve trunks are known to traverse, and have significant associations with the psoas major and iliopsoas muscles (13). Our research corroborates that these tumors, irrespective of their classification as classic, cell-rich, or malignant, exhibit a heightened predilection for the paraspinal region of the abdominopelvic cavity, thereby reinforcing the nexus between the sites of prevalence and nerve distribution.

The mean age of onset for malignant retroperitoneal nerve sheath tumors in our study was significantly lower, a finding that is congruent with existing literature [14]. Moreover, the presence of a history of nerve sheath tumors or comorbid neurofibromatosis in some patients within the malignant cohort mirrors observations from prior studies [15]. In stark contrast, the majority of classic and cell-rich nerve sheath tumors were found to be solitary, aligning with earlier reports [16].

The lax anatomical structure of retroperitoneal space permits nerve sheath tumors to attain considerable sizes, often asymptomatic, until they reach a substantial magnitude. Classic and cell-rich nerve sheath tumors may exhibit hemorrhage or cystic degeneration, with the cell-rich variant being more prone to tissue invasion and recurrence than its classic counterpart [17].

Sonographically, cell-rich nerve sheath tumors tend to display chaotic internal echoes and heightened vascularity, even though no statistically significant differences were noted between the two in terms of tissue invasiveness. Pathologically, these tumors are distinguished by the presence of Antoni A and B regions, with Antoni A representing the solid, highly vascularized component of the lesion [18]. Classical schwannomas often consist of alternating fascicular areas (Antoni A) and reticular areas (Antoni B), which may either transition smoothly or show clear boundaries. Antoni A is composed of bundles of parallel-arranged Schwann cells, which are dense and cellular, with indistinct borders, no atypia, occasional mitoses, and numerous small blood vessels. Antoni B consists of loosely arranged, disorganized Schwann cells, with hyalinized blood vessels visible. In contrast, cellular schwannomas are characterized by uniform cell morphology, with tightly bundled or interwoven patterns resembling Antoni A areas of classic schwannomas but with limited or only localized Antoni B areas, showing mild to moderate cellular heterogeneity [19]. These tumors have thicker-walled vessels compared to classical schwannomas, accompanied by perivascular lymphocytic infiltration [19]. The ultrasound characteristics in our study revealed notable differences, with malignant tumors presenting a larger mean maximum diameter compared to classic and cell-rich tumors (*p*=0.027) and malignant tumors exhibiting atypical features, including indistinct borders (*p*=0.000) and a predominance of irregular shapes (*p*=0.001). However, malignant tumors showed significantly less distinct delineation from surrounding tissues (*p*=0.000), further reflecting their invasive nature.

Ultrasound serves as an invaluable initial assessment and monitoring tool for Retroperitoneal Benign Nerve Sheath Tumors (RBNST), particularly for small lesions or in non-invasive settings [1]. Malignant retroperitoneal nerve sheath tumors often present as larger masses, as evidenced by the maximum diameter cutoff value exceeding 9.9 cm and an AUC of 0.754 in our study. The possibility of malignancy should be contemplated when the tumor's maximum diameter surpasses 10 cm. These tumors frequently exhibit blurred borders and irregular morphology on ultrasound, indicative of their invasive nature and consistent with their classification as high-grade malignant soft tissue sarcomas [20]. The surrounding soft tissue reaction in malignant nerve sheath tumors often results in irregular, hyperechoic margins and uneven internal echoes, augmenting their metastatic potential [21].

While ultrasonography may have limitations in differentiating between classic and cell-rich nerve sheath tumors, it adeptly delineates the tumor's location and its relationship with peripheral nerves, which is invaluable for the differential diagnosis of malignant nerve sheath tumors. Jin *et al*. showed that a combination of clinical diagnosis and sonography can achieve the same effect as MRI for diagnosing peripheral nerve sheath tumors [22]. Furthermore, this may also help in developing AI-based models to differentiate between classic and cell-rich nerve sheath tumors, as similar technology for the diagnosis of various medical conditions has yielded promising results [23-27].

Accurate diagnosis is imperative for these highly aggressive tumors, assisting clinicians in focusing on the disease and circumventing potential surgical complications, such as nerve damage.

The ultrasonographic features observed in our study, including irregular borders, indistinct margins, and larger tumor size in malignant nerve sheath tumors, may be used as non-invasive markers of aggressive biological behavior as these align with the pathological findings of increased tissue invasion and recurrence risk in malignant variants. The identification of such features during ultrasound examinations may guide clinical decisions, prompting more frequent follow-up, advanced imaging, or even early surgical intervention. However, the lack of significant differences in echogenicity or calcification suggests that sonography alone may not fully capture the complexity of tumor biology. Therefore, a multidisciplinary approach is required, incorporating clinical exams and histopathology. It is important to acknowledge the limitations of our study. Moreover, a relatively small sample size, particularly for malignant tumors, may affect the generalizability of the results. Ultrasonography also has several limitations, including operator dependency, narrow field of view, and limited tissue characterization. Furthermore, the retrospective nature of this study may introduce selection bias.

Future research endeavors should aim to expand the sample size and incorporate additional clinical and pathological parameters to provide a more holistic understanding of the characteristics and treatment strategies for retroperitoneal nerve sheath tumors.

## CONCLUSION

Retroperitoneal nerve sheath tumors present with a spectrum of clinical and sonographic attributes that are especially pronounced in malignant cases. As a diagnostic modality, ultrasonography offers clarity in tumor classification and informs tailored treatment approaches. Our findings highlight the significance of clinical features and ultrasound manifestations in diagnosing retroperitoneal nerve sheath tumors.

This study has several limitations, including a small sample size, ultrasonography's operator dependency, and retrospective design.

The ultrasound features of malignant nerve sheath tumors, such as unclear borders and irregular morphology, may be correlated with their heightened invasiveness and malignant potential. For tumors with atypical ultrasound presentations, further imaging and pathological analysis are advised to ensure precise diagnosis and treatment.

Future research should aim to expand the sample size and incorporate additional clinical and pathological parameters to better understand retroperitoneal nerve sheath tumors.

## Figures and Tables

**Fig. (4):**
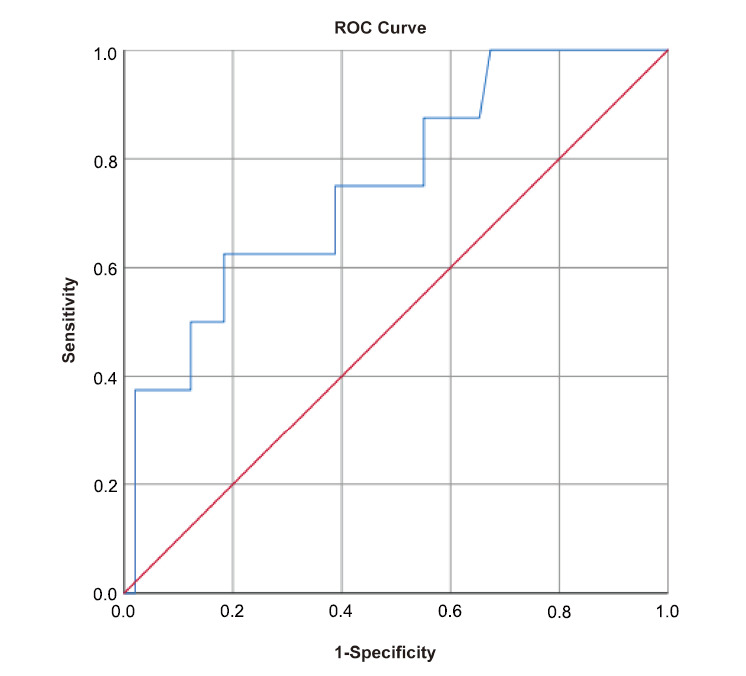
ROC curve analysis for the maximum diameter of tumors.

**Table 1:** Comparison of clinical data among the three groups (Kruskal-Wallis test).

Clinical Information	Classic Nerve Sheath Tumor (n=36)	Cell-rich Nerve Sheath Tumor (n=13)	Malignant Nerve Sheath Tumor (n=8)	χ^2^	*p*-value
Age (years)	42.5(28-58)	56(34.5-61.5)	40.5(33.5-45.75)	3.525	0.172
**Sex**	-	-	-	2.660	0.273
Male	12 (33.3%)	4 (30.8%)	5 (62.5%)	-	-
Female	24 (66.7%)	9 (69.25)	3 (37.5%)	-	-
**Number of tumors**	-	-	-	14.118	**0.004**
Single	36 (100%)	12 (92.3%)	5 (62.5%)	-	-
Multiple	0 (zero)	1 (7.7%)	3 (37.5%)	-	-
**Location**	-	-	-	6.612	0.113
Abdominal	20 (55.6%)	5 (38.5%)	4 (50.0%)	-	-
Pelvic	15 (41.7%)	5 (38.5%)	2 (25.0%)	-	-
**Abdominal and pelvicc**	1 (277%)	3 (23.0%)	2 (25.0%)	-	-

**Table 2:** Comparison of ultrasound characteristics between the three groups.

Ultrasonic Characterization	Classic Nerve Sheath Tumor (n=36)	Cell-rich Nerve Sheath Tumor (n=13)	Malignant Nerve Sheath Tumor (n=8)	χ^2^	*p*-value
Maximum Diameter (cm)	7.3(5.23-9.35)	7.9(4.7-12.75)	11.45(7.3-15.75)	5.833	0.054
**Frontier**	-	-	-	19.264	**0.000**
Clear	34 (94.4%)	13 (100%)	2 (25%)	-	-
Ambiguous	2 (5.6%)	0 (zero)	6 (75%)	-	-
**Morphological**	-	-	-	12.729	**0.001**
Regular	34 (94.4%)	10 (76.9%)	3 (37.5%)	-	-
Irregular	2 (5.6%)	3 (23.1%)	5 (62.5%)	-	-
**Internal Echo**	-	-	-	6.197	0.115
Low echo	34 (94.4%)	11 (84.6%)	6 (75%)	-	-
Isochronous	1 (2.8%)	0 (zero)	0 (zero)	-	-
Low echo predominantly, with iso-echo	1 (2.8%)	2 (15.4%)	2 (25%)	-	-
**Ingredient**	-	-	-	3.485	0.770
Solid	17 (47.2%)	4 (30.8%)	3 (37.5%)	-	-
Cystic solidity	3 (8.3%)	3 (23.1%)	1 (12.5%)	-	-
Cystic solid, mainly cystic	2 (5.6%)	1 (7.7%)	0 (zero)	-	-
Cystic solid, mainly solid	14 (38.9%)	5 (38.5%)	4 (50.0%)	-	-
**Calcification**	-	-	-	1.192	0.765
Yes	2 (5.6%)	1 (7.7%)	1 (12.5%)	-	-
No	34 (94.4%)	12 (92.3%)	7 (87.5%)	-	-
**Boundary with surrounding organizations**	-	-	-	25.014	**0.000**
Clear	36 (100%)	13 (100%)	2 (25%)	-	-
Ambiguous	0 (zero)	0 (zero)	6 (75%)	-	-
Bood flow classification	-	-	-	6.379	0.355
0	18 (50.0%)	3 (23.1%)	2 (25.0%)	-	-
1	7 (19.4%)	6 (46.2%)	2 (25.0%)	-	-
2	513.9%)	1 (7.7%)	2 (25.0%)	-	-
3	6 (16.7%)	3 (23.1%)	2 (25.0%)	-	-

**Table 3:** Multiple regression results.

Group^a^		B	Standard Error	Wald	Significance	Exp(B)	Exp(B) 95% Confidence Interval of Exp(B)	
							Lower Limit	Upper Limit
Highly Cellular	Intercept	-27.608	2444.306	0.000	0.991	-	-	-
	Gender	0.811	0.830	0.954	0.329	2.250	0.442	11.446
	Age	0.043	0.027	2.505	0.113	1.044	0.990	1.100
	Number	41.934	2445.010	0.000	0.986	1627809954789159940.000	.000	.^b^
	Maximum Diameter	0.006	0.102	0.003	0.954	1.006	0.823	1.229
	Boundary	-42.600	2412.518	0.000	0.986	3.155E-19	0.000	.^b^
	Morphology	2.590	1.567	2.732	0.098	13.328	0.618	287.465
	Relationship with Surrounding Tissue	20.964	0.000	.	.	1272090513.808	1272090513.808	1272090513.808
Malignant	Intercept	-290.873	6349.000	0.002	0.963	-	-	-
	Gender	43.051	1057.341	0.002	0.968	4976980293477489700.000	0.000	.^b^
	Age	-.667	27.784	0.001	0.981	0.513	1.149E-24	229139161479969750000000.000
	Number	82.288	2550.677	0.001	0.974	546031494922585500000000000000000000.000	.000	.^b^
	Maximum Diameter	4.285	96.121	0.002	0.964	72.630	1.104E-80	4.779E+83
	Boundary	10.636	1109.132	0.000	0.992	41611.565	0.000	.^b^
	Morphology	10.525	715.864	0.000	0.988	37228.710	0.000	.^b^
	Relationship with Surrounding Tissue	61.946	3466.378	0.000	0.986	799846304024394600000000000.000	0.000	.^b^

Note: *Due to the small sample size, some groups have values of zero in certain categories after classification, resulting in unsatisfactory outcomes.

## Data Availability

All the data and supporting information are provided within the article.

## LIST OF ABBREVIATIONS

MPNSTs: Malignant Peripheral Nerve Sheath Tumors
RBNSTs: Retroperitoneal Benign Nerve Sheath Tumors
US: Ultrasound
ROC: Receiver Operating Characteristic
AUC: Area Under the Curve

## STANDARDS OF REPORTING

STROBE guidelines were followed.
